# Drug-resistant tuberculosis in Lishui, China: first-line drug resistance patterns, trends, and risk factors from a 10-year retrospective study (2015–2024)

**DOI:** 10.3389/fpubh.2026.1855459

**Published:** 2026-06-17

**Authors:** Honghui Yang, Yutong Zhang, Henan Xu, Xueling Li, Chenbo Zhou, Congjuan Lai

**Affiliations:** Department of Clinical Laboratory, Lishui Traditional Chinese Medicine Hospital, Lishui, Zhejiang, China

**Keywords:** drug resistance, Lishui, multidrug-resistant tuberculosis, risk factors, tuberculosis

## Abstract

**Purpose:**

Despite ongoing global efforts, drug-resistant TB (DR-TB) continues to pose a substantial threat to public health, and systematic longitudinal surveillance data on local resistance patterns are lacking in many regions. This study analyzed first-line drug resistance patterns and annual trends in Lishui, China from 2015 to 2024 and identified independent risk factors for multidrug-resistant tuberculosis (MDR-TB).

**Methods:**

A total of 2,339 TB patients with available phenotypic drug susceptibility testing (pDST) results from the designated TB treatment institution in Lishui were retrospectively enrolled between January 2015 and December 2024. Annual drug resistance trends were assessed, and independent risk factors for MDR-TB were evaluated through multivariate logistic regression.

**Results:**

The overall prevalence of DR-TB and MDR-TB was 20.09 and 6.93%, respectively. Over the 10-year period, overall DR-TB prevalence declined from 23.50% in 2015 to 15.63% in 2024 (APC: −1.30%, *p* = 0.661), with this reduction largely attributable to the declining trend among retreated patients (56.25–15.00%, APC: −8.19%, *p* = 0.156), while resistance rates in new patients remained broadly stable (APC: +0.95%, *p* = 0.732). MDR-TB prevalence in retreated patients decreased significantly from 40.63 to 15.00% (Cochran-Armitage trend test, *p* = 0.008). Prior TB treatment history (AOR = 4.345, 95% CI: 3.029–6.234, *p* < 0.001) and farmer occupation (AOR = 1.638, 95% CI: 1.047–2.562, *p* = 0.031) were identified as independent risk factors for MDR-TB.

**Conclusion:**

The decrease in drug resistance among retreated patients was the main driver of the overall decline in DR-TB prevalence observed in Lishui across the study period. The relative stability of resistance in new patients suggests ongoing circulation of drug-resistant strains in the community, highlighting the need for expanded drug susceptibility testing (DST) coverage and strengthened community infection control. Enhanced treatment adherence and universal pDST coverage, particularly for retreated patients and socioeconomically vulnerable populations such as farmers, are essential for effective MDR-TB containment.

## 1 Introduction

Tuberculosis remains a preeminent infectious cause of mortality globally, exhibiting a fatality rate approximately double the rate for HIV/AIDS, thereby posing a persistent and severe danger to global population health. The Global Tuberculosis Report 2025 estimated that approximately 1.23 million individuals died from TB in 2024. Between 2015 and 2024, TB-related deaths declined by only 29% globally, falling far short of the 75% reduction target set for 2025, indicating that global TB control remains a major challenge (1). Simultaneously, the occurrence and spread of drug-resistant TB (DR-TB) have intensified this crisis. Specifically, MDR-TB, which is defined by resistance to both isoniazid and rifampicin, is associated with limited treatment options, substantially prolonged treatment duration, significantly increased costs, and higher rates of treatment failure and mortality, imposing a considerable burden on both patients and society (2, 3).

The Global Tuberculosis Report 2025 estimated that around 400,000 individuals developed MDR/Rifampicin-resistant tuberculosis (RR-TB) globally in 2024, constituting 3.2% of incident TB cases and 16% of retreatment cases (1). As a high-burden country, China estimated 28,000 new MDR/RR-TB cases in 2024; notably, the proportion among previously treated patients (18.7%) was substantially higher than among new cases (2.9%), underscoring the formidable challenges in local resistance containment (4). The pathogenesis of MDR-TB involves two distinct mechanisms: acquired resistance, where suboptimal treatment regimens or poor adherence facilitates the selective enrichment of resistant strains under antimicrobial pressure (5); and primary resistance, arising from the direct dissemination of resistant strains within communities (6). Prior TB treatment history is widely recognized as the most important risk factor for MDR-TB, and retreated patients carry a significantly higher risk of drug resistance compared with new patients (7).

While several provincial and city-level drug resistance surveillance studies have been conducted in China (8–12), resistance profiles and epidemic trends vary considerably across regions due to differences geographic, demographic, and socioeconomic conditions (1). This heterogeneity underscores the critical importance of localized surveillance in informing targeted prevention and control strategies. Lishui, a mountainous city in southwestern Zhejiang Province with a permanent population of approximately 2.7 million, faces unique challenges in TB control due to its agriculturally dominant population and frequent migration. Since 2015, the continuous implementation of the Directly Observed Treatment, Short-course (DOTS) strategy and dedicated DR-TB programs, alongside the progressive expansion of drug susceptibility testing (DST) coverage, has established a foundation for longitudinal surveillance in Lishui. However, no systematic study with a broad time span has examined TB drug resistance patterns and trends in Lishui, and the local resistance profile and its associated factors remain unclear. The present research aimed to retrospectively evaluate the characteristics and annual dynamics of first-line drug resistance among TB cases within Lishui between 2015 and 2024, as well as to determine independent predictors for MDR-TB, serving the goal of providing evidence-based guidance for local anti-TB drug procurement, treatment regimen optimization, and DR-TB containment and management protocols.

## 2 Materials and methods

### 2.1 Setting and participants

This study was conducted in Lishui (27°25′–28°57′N, 118°41′–120°26′E), a mountainous city in southwestern Zhejiang Province, China. Covering a total area of 17,300 km^2^, the city comprises nine counties and districts with a permanent population of 2.532 million as of December 2024. The investigation was performed at Lishui Municipal Hospital of Traditional Chinese Medicine, the sole designated municipal-level institution for TB diagnosis and treatment in Lishui, to which culture-positive cases from across the region are referred for pDST.

Patient records containing sociodemographic profiles (sex, ethnicity, age, occupation, residence, and hukou type), pDST findings, and treatment history were sourced from TBIMS and the corresponding hospital laboratory database.

**Figure 1:**
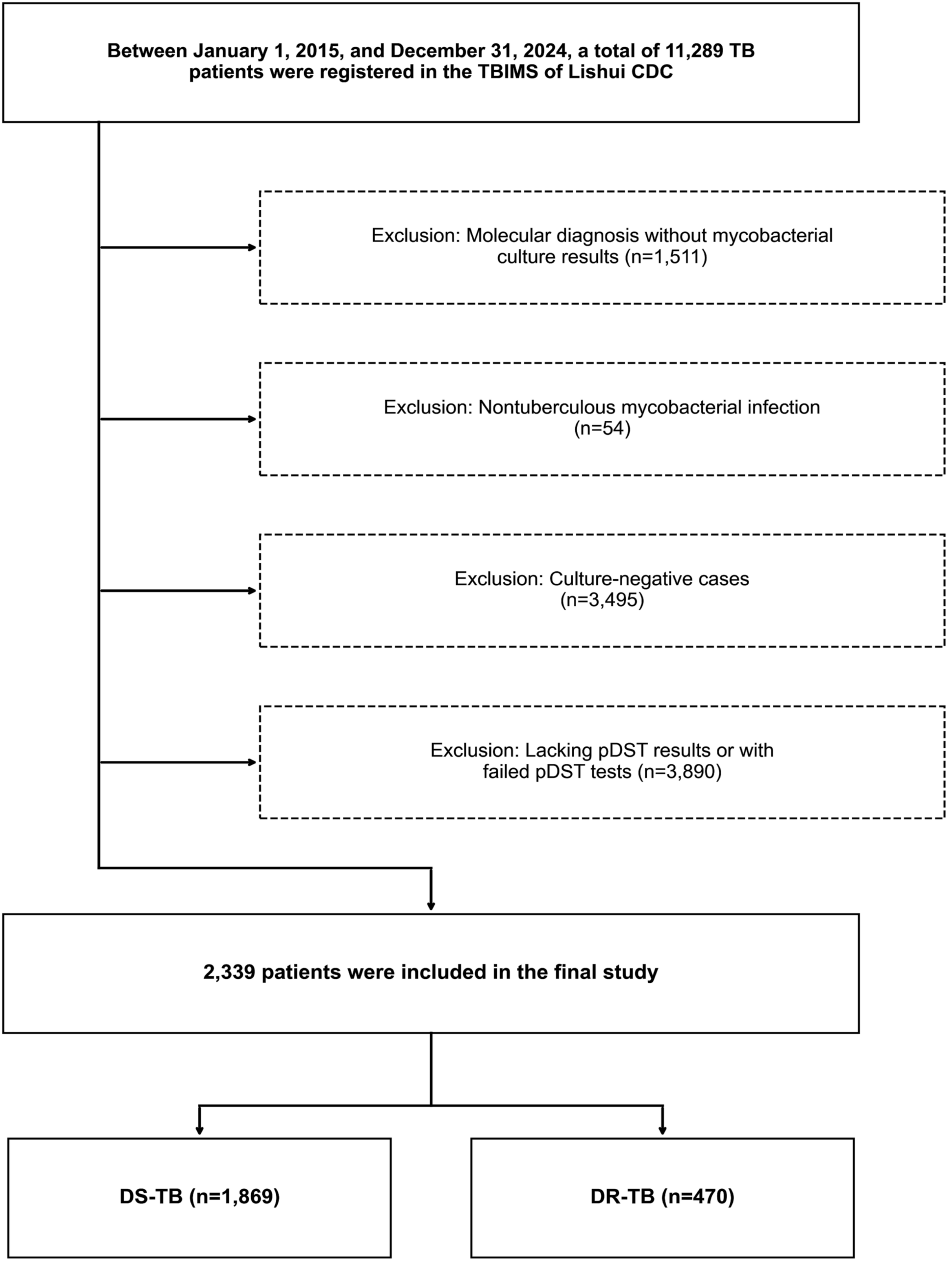
Flow diagram detailing the participant screening process.

Between Jan 2015 and Dec 2024, an overall 11,289 confirmed TB records were documented within the Lishui Centers for Disease Control and Prevention (CDC) database. Of these, 1,511 cases were excluded due to reliance on molecular diagnosis alone lacking concurrent mycobacterial culture data. Furthermore, 54 patients recognized to have non-tuberculous mycobacterial (NTM) infections were omitted, alongside 3,495 culture-negative cases and 3,890 cases with missing or failed pDST results. Ultimately, 2,339 eligible patients were retained for the final evaluation (Figure 1).

### 2.2 Definitions

DR-TB was defined as resistance to one or more anti-TB drugs. MDR-TB was defined as resistance to at least both isoniazid and rifampicin. For descriptive purposes, mono-resistant TB (MR-TB) was defined as resistance to a single first-line drug, and non-MDR polydrug-resistant TB (PDR-TB) was defined as resistance to two or more first-line drugs in the absence of concurrent resistance to both isoniazid and rifampicin; PDR-TB as used here should not be confused with pandrug-resistant TB. As susceptibility testing in this study was restricted to four first-line drugs and did not include fluoroquinolones, bedaquiline, or linezolid, pre-XDR-TB and XDR-TB could not be assessed; accordingly, MDR-TB cases in this study may include patients who would meet criteria for pre-XDR-TB or XDR-TB if second-line DST were available (13).

New patients were defined as those meeting any of the following criteria: no prior anti-TB treatment; currently receiving a standard chemotherapy regimen but having completed less than a full course; or having received unsystematic anti-TB treatment for fewer than 30 days. Retreated patients were defined as those meeting any of the following criteria: prior use of anti-TB drugs for an inadequate or irregular regimen for 1 month or more; or patients with initial treatment failure or relapse. Migrants were defined as individuals residing in a location other than their hukou-registered township or street-level address for a continuous period of 6 months or longer. Occupational information was extracted from TBIMS registry records, where occupation was reported by patients at registration. The “farmer” category encompasses individuals engaged in agricultural production as their primary occupation.

### 2.3 Drug susceptibility testing

Respiratory specimens, including sputum and bronchoalveolar lavage fluid, collected at admission were cultured using BACTEC MGIT liquid medium (BD, United States) or LJ solid medium for culture. Positive cultures were confirmed as acid-fast bacilli via smear microscopy and identified as *Mycobacterium tuberculosis* (MTB) using MPB64 antigen detection, followed by phenotypic drug susceptibility testing (pDST) via the MGIT liquid method. The critical concentrations for the four first-line drugs were 1.0 μg/mL for streptomycin (STR), 0.1 μg/mL for isoniazid (INH), 1.0 μg/mL for rifampicin (RIF), and 5.0 μg/mL for ethambutol (EMB). Although streptomycin is no longer classified as a first-line anti-tuberculosis drug by the World Health Organization, it remains categorized as a first-line drug in China’s national TB prevention and control guidelines (2020 edition) (14) and continues to be routinely tested in clinical drug susceptibility testing in China. Laboratory quality control was maintained through external quality assessment conducted regularly by the National TB Reference Laboratory of the China CDC, and internal quality control was performed monthly using the H37Rv reference strain.

### 2.4 Statistical analysis

All data were managed and organized using WPS Excel (v12.1.0.24034, Kingsoft Office Software, Beijing, China) and drug resistance trend figures were generated using Python (Matplotlib library). All statistical analyses were conducted with SPSS v31.0 (IBM Corp., Armonk, NY, United States). Categorical variables were summarized as frequencies and proportions, with between-group comparisons performed using Pearson’s *χ*^2^ test or the continuity-corrected *χ*^2^ test. Joinpoint regression analysis was performed using the NCI Joinpoint Regression Program to evaluate temporal trends in overall DR-TB prevalence and quantify the Annual Percent Change (APC) with 95% confidence intervals; model selection was based on the Permutation Test (overall significance level 0.05, maximum 4,499 permutations), with a maximum of two joinpoints allowed for total and new cases, and one joinpoint for retreated cases. The Cochran-Armitage trend test was applied to assess temporal trends in individual first-line anti-tuberculosis drug resistance rates and in the annual proportions of drug resistance categories, including MDR-TB, PDR-TB, and MR-TB, stratified by patient group. Univariate logistic regression was employed to examine patient demographic characteristics, and Variables achieving *p* < 0.05 in univariate screening, together with sex and age as *a priori* clinically relevant covariates, were subsequently included in a multivariate logistic regression model using the forced entry method to determine independent predictors of MDR-TB. A two-tailed *p* < 0.05 was adopted as the threshold for statistical significance throughout.

## 3 Results

### 3.1 Patient characteristics

Between 2015 and 2024, 11,289 confirmed TB cases were registered in Lishui. Of these, 9,724 underwent mycobacterial culture, with an overall positivity rate of 64.06%. Annual culture positivity rates increased from 59.40% (2015) to 72.05% (2024). pDST was performed on 2,339 isolates, representing 37.55% of all culture-positive cases. Annual pDST coverage declined from 54.35% (2015) to 37.30% (2024), with the lowest rate (32.29%) recorded in 2021 during the COVID-19 pandemic (Supplementary Table S1).

Among the 2,339 TB patients included in this study from 2015 to 2024, 1,869 (79.91%) were diagnosed with drug-susceptible TB (DS-TB) and 470 (20.09%) with drug-resistant TB (DR-TB). Among all patients, 1,765 (75.46%) were male and 574 (24.54%) were female. Regarding age distribution, the 60–74 years group represented the largest cohort (30.14%), followed by the 18–44 (25.52%) and 45–59 (24.71%) years groups. Most patients were of Han ethnicity (98.42%), and 492 (21.03%) were identified as migrants. A total of 2,071 patients (88.54%) were new cases. Farmers represented the predominant occupational group (71.91%).

The proportion of retreated patients was significantly higher in the DR-TB group than in the DS-TB group (19.36% vs. 9.47%, *p* < 0.001). The DR-TB group also had slightly higher proportions of male patients, those aged 60–74 years, migrants, and farmers compared with the DS-TB group, but these differences were not statistically significant (Table 1).

**Table 1:** Sociodemographic characteristics of TB patients.

Characteristics		Total, *n* = 2,339 (%)	DS-TB, *n* = 1869 (%)	DR-TB, *n* = 470 (%)	*P*-value
Gender	Male	1765(75.46%)	1,397(74.75%)	368(78.30%)	0.110
	Female	574(24.54%)	472(25.25%)	102(21.70%)	
Age (years)	<18	54(2.31%)	46(2.46%)	8(1.70%)	0.599
	18–44	597(25.52%)	483(25.84%)	114(24.26%)	
	45–59	578(24.71%)	465(24.88%)	113(24.04%)	
	60–74	705(30.14%)	551(29.48%)	154(32.77%)	
	≥75	405(17.32%)	324(17.34%)	81(17.23%)	
Ethnicity	Han	2,302(98.42%)	1837(98.29%)	465(98.94%)	0.314
	Others	37(1.58%)	32(1.71%)	5(1.06%)	
Migrant	Yes	492(21.03%)	391(20.92%)	101(21.49%)	0.787
	No	1847(78.97%)	1,478(79.08%)	369(78.51%)	
Patient category	New cases	2071(88.54%)	1,692(90.53%)	379(80.64%)	<0.001
	Retreated cases	268(11.46%)	177(9.47%)	91(19.36%)	
Occupation	Farmer	1,682(71.91%)	1,333(71.32%)	349(74.26%)	0.206
	Others	657(28.09%)	536(28.68)	121(25.74%)	

To assess potential selection bias introduced by the exclusion of patients with missing or failed pDST results, we compared the baseline sociodemographic characteristics of the included cohort (*n* = 2,339) with those of the pDST-missing group (*n* = 3,890) (Supplementary Figure S1). The two groups showed no significant differences in the distributions of sex (75.5% vs. 74.1% male, *p* = 0.258), age (*p* = 0.465), occupation (71.9% vs. 72.3% farmers, *p* = 0.770), or ethnicity (98.4% vs. 99.4% Han, *p* < 0.001). However, the included cohort had a significantly higher proportion of retreated patients (11.5% vs. 8.4%, *p* < 0.001) and migrants (21.0% vs. 11.5%, *p* < 0.001) compared with the pDST-missing group.

### 3.2 Comparison of drug resistance patterns between new and retreated patients

Among the 2,071 new patients, resistance rates to the four first-line drugs were highest for INH (12.07%), followed by STR (11.15%), RIF (6.28%), and EMB (3.67%). For the 268 retreated patients, resistance rates were significantly higher across all four drugs: INH (25.75%), RIF (24.63%), STR (20.90%), and EMB (10.82%). The resistance ranking shifted to INH > RIF > STR > EMB in retreated patients, with significantly higher rates compared to new patients for each drug (*p* < 0.001).

For resistance categories, new patients had significantly lower proportions of DR-TB (18.30% vs. 33.96%) and MDR-TB (5.21% vs. 20.15%) than retreated patients (both *p* < 0.001). The proportions of MR-TB (10.00% vs. 10.07%, *p* = 0.967) and PDR-TB (3.09% vs. 3.73%, *p* = 0.573) were similar between the two groups. In MDR-TB patterns, INH + RIF + STR and INH + RIF + STR + EMB were the most frequent combinations in both groups, though their proportions were significantly higher in retreated patients (both 7.46%) than in new patients (2.03 and 1.98%, respectively; both *p* < 0.001) (Table 2).

**Table 2:** Drug resistance patterns in new and retreated TB patients.

Drug resistance pattern	New case, *n* = 2071(%)	Retreated case, *n* = 268(%)	*P*-value
DR-TB	379(18.30%)	91(33.96%)	<0.001
Any resistance			
Any INH	250(12.07%)	69(25.75%)	<0.001
Any RIF	130(6.28%)	66(24.63%)	<0.001
Any STR	231(11.15%)	56(20.90%)	<0.001
Any EMB	76(3.67%)	29(10.82%)	<0.001
MR-TB	207(10.00%)	27(10.07%)	0.967
INH	85(4.10%)	9(3.36%)	0.558
RIF	17(0.82%)	8(3.0%)	0.006
STR	90(4.3%)	8(2.99%)	0.296
EMB	15(0.72%)	2(0.75%)	0.968
MDR-TB	108(5.21%)	54(20.15%)	<0.001
INH + RIF	19(0.92%)	11(4.10%)	<0.001
INH + RIF + STR	42(2.03%)	20(7.46%)	<0.001
INH + RIF + EMB	6(0.29%)	3(1.12%)	0.082
INH + RIF + STR + EMB	41(1.98%)	20(7.46%)	<0.001
Non-MDR polydrug-resistant TB (PDR-TB)^a^	64(3.09%)	10(3.73%)	0.573
RIF + STR	4(0.19%)	2(0.75%)	0.092
RIF + EMB	0(0.00%)	1(0.37%)	0.005
INH + STR	46(2.22%)	4(1.49%)	0.438
INH + EMB	6(0.29%)	1(0.37%)	0.814
STR + EMB	2(0.10%)	0(0.00%)	0.611
STR + EMB + INH	5(0.24%)	1(0.37%)	0.688
STR + EMB + RIF	1(0.05%)	1(0.37%)	0.087

^a^Refers to resistance to two or more first-line drugs in the absence of concurrent resistance to both isoniazid and rifampicin.

### 3.3 Annual trends in overall drug resistance rates, 2015–2024

The overall prevalence of DR-TB in Lishui was 23.50% in 2015 and 15.63% in 2024. Joinpoint regression analysis identified no statistically significant joinpoints over the study period (Final Selected Model: 0 joinpoints), with an overall APC of −1.30% (95% CI: −9.56 to 7.42, *p* = 0.661). A transient increase in overall DR-TB prevalence was observed in 2021 (23.61%) and 2022 (30.10%), before declining to 19.23% in 2023 and 15.63% in 2024. Of note, 2021 recorded the lowest total case count in the study period (*n* = 144), with only 17 retreated patients.

**Figure 2:**
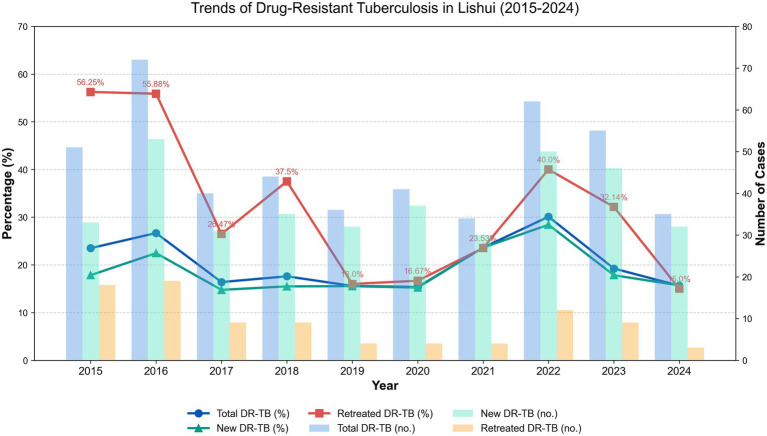
Annual trends in the number and proportion of drug-resistant TB cases stratified by treatment history, Lishui, China, 2015–2024. Bar charts (right *Y*-axis) indicate the annual number of total, new, and retreated DR-TB cases; line graphs (left *Y*-axis) indicate the corresponding annual DR-TB proportions among total, new, and retreated patients.

Stratified analysis by treatment history revealed that among new patients, although the prevalence at the end of the study period (15.69% in 2024) was lower than at baseline (17.84% in 2015), joinpoint regression indicated a marginal non-significant upward trend over the entire study period (APC: +0.95%, 95% CI: −4.80 to 7.01, *p* = 0.732). Among retreated patients, DR-TB prevalence was 56.25% in 2015 and 15.00% in 2024, with a non-significant downward trend observed (APC: −8.19%, 95% CI: −23.91 to 5.14, *p* = 0.156). Details are presented in Table 3 and Figure 2.

**Table 3:** Annual trends in drug-resistant TB prevalence stratified by treatment history.

Year	Total case	DR-TB, *n* (%)	New case	DR-TB, *n* (%)	Retreated case	DR-TB, *n* (%)
2015	217	51 (23.50%)	185	33 (17.84%)	32	18 (56.25%)
2016	270	72 (26.67%)	236	53 (22.46%)	34	19 (55.88%)
2017	244	40 (16.39%)	210	31 (14.76%)	34	9 (26.47%)
2018	250	44 (17.60%)	226	35 (15.49%)	24	9 (37.50%)
2019	231	36 (15.58%)	206	32 (15.53%)	25	4 (16.00%)
2020	267	41 (15.36%)	243	37 (15.23%)	24	4 (16.67%)
2021	144	34 (23.61%)	127	30 (23.62%)	17	4 (23.53%)
2022	206	62 (30.10%)	176	50 (28.41%)	30	12 (40.00%)
2023	286	55 (19.23%)	258	46 (17.83%)	28	9 (32.14%)
2024	224	35 (15.63%)	204	32 (15.69%)	20	3 (15.00%)
APC		−1.30%		+0.95%		−8.19%
95% CI		(−9.56, 7.42)		(−4.80, 7.01)		(−23.91, 5.14)
P-value		0.661		0.732		0.156
Final selected model		0		0		0
Total	2,339		2071		268	

### 3.4 Annual trends in first-line drug resistance among new and retreated patients

Cochran-Armitage trend analysis identified a significant decrease in STR resistance rate among new patients, from 11.35 to 5.88% (*χ*^2^ = 5.019, *p* = 0.025). Although resistance rates at the end of the study period were numerically lower than at baseline for INH (12.97% vs. 12.25%), RIF (9.73% vs. 4.41%), and EMB (7.03% vs. 0.49%), no statistically significant linear trends were detected for these three drugs (INH: *χ*^2^ = 0.845, *p* = 0.358; RIF: *χ*^2^ = 0.422, *p* = 0.516; EMB: *χ*^2^ = 0.280, *p* = 0.597).

**Figure 3:**
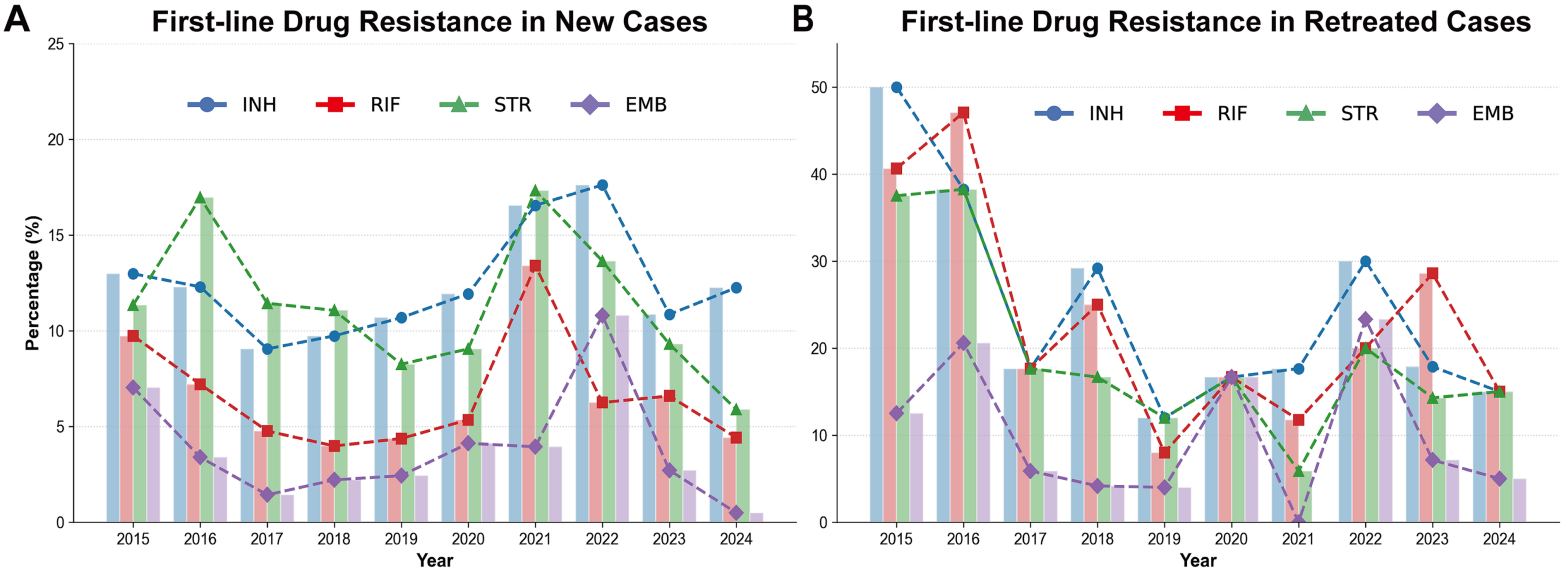
Annual trends in the proportions of first-line drug resistance among new and retreated TB patients, Lishui, China, 2015–2024. **(A)** Annual changes in INH, RIF, STR, and EMB resistance proportions among new TB patients. **(B)** Corresponding resistance patterns among retreated TB patients.

Resistance rates for all four first-line drugs showed more substantial declines in retreated patients. Significant downward trends were observed for INH (50.00 to 15.00%, *χ*^2^ = 8.160, *p* = 0.004), RIF (40.63 to 15.00%, *χ*^2^ = 6.948, *p* = 0.008), and STR (37.50 to 15.00%, *χ*^2^ = 8.263, *p* = 0.004). The EMB resistance rate decreased from 12.50 to 5.00%, though this trend was not statistically significant (*χ*^2^ = 0.298, *p* = 0.585) (Table 4 and Figure 3).

**Table 4:** Annual trends in first-line anti-tuberculosis drug resistance rates among new and retreated patients.

Year	New case, *n* (%)					Retreated case, *n* (%)				
	No.	INH	RIF	STR	EMB	No.	INH	RIF	STR	EMB
2015	185	24 (12.97%)	18 (9.73%)	21 (11.35%)	13 (7.03%)	32	16 (50.00%)	13 (40.63%)	12 (37.50%)	4 (12.50%)
2016	236	29 (12.29%)	17 (7.20%)	40 (16.95%)	8 (3.39%)	34	13 (38.24%)	16 (47.06%)	13 (38.24%)	7 (20.59%)
2017	210	19 (9.05%)	10 (4.76%)	24 (11.43%)	3 (1.43%)	34	6 (17.65%)	6 (17.65%)	6 (17.65%)	2 (5.88%)
2018	226	22 (9.73%)	9 (3.98%)	25 (11.06%)	5 (2.21%)	24	7 (29.17%)	6 (25.00%)	4 (16.67%)	1 (4.17%)
2019	206	22 (10.68%)	9 (4.37%)	17 (8.25%)	5 (2.43%)	25	3 (12.00%)	2 (8.00%)	3 (12.00%)	1 (4.00%)
2020	243	29 (11.93%)	13 (5.35%)	22 (9.05%)	10 (4.12%)	24	4 (16.67%)	4 (16.67%)	4 (16.67%)	4 (16.67%)
2021	127	21 (16.54%)	17 (13.39%)	22 (17.32%)	5 (3.94%)	17	3 (17.65%)	2 (11.76%)	1 (5.88%)	0 (0.00%)
2022	176	31 (17.61%)	11 (6.25%)	24 (13.64%)	19 (10.80%)	30	9 (30.00%)	6 (20.00%)	6 (20.00%)	7 (23.33%)
2023	258	28 (10.85%)	17 (6.59%)	24 (9.30%)	7 (2.71%)	28	5 (17.86%)	8 (28.57%)	4 (14.29%)	2 (7.14%)
2024	204	25 (12.25%)	9 (4.41%)	12 (5.88%)	1 (0.49%)	20	3 (15.00%)	3 (15.00%)	3 (15.00%)	1 (5.00%)
	2071	250 (12.07%)	130 (6.28%)	231 (11.15%)	76 (3.67%)	268	69 (25.75%)	66 (24.63%)	56 (20.90%)	29 (10.82%)
*χ* ^2^		0.845	0.422	5.019	0.280		8.160	6.948	8.263	0.298
*P*-value		0.358	0.516	0.025	0.597		0.004	0.008	0.004	0.585

### 3.5 Temporal patterns of drug resistance categories in new and retreated TB patients

In new patients, the proportions of MR-TB, PDR-TB, and MDR-TB were 7.57%, 1.62%, and 8.65% in 2015, and 11.27%, 0.98%, and 3.43% in 2024, respectively. None of these endpoint differences constituted a statistically significant linear trend over the study period (MR-TB: *χ*^2^ = 0.918, *p* = 0.338; PDR-TB: *χ*^2^ = 0.359, *p* = 0.549; MDR-TB: *χ*^2^ = 0.937, *p* = 0.333) by Cochran-Armitage trend test, and no consistent directional shift in resistance category composition was identified among new patients.

**Figure 4:**
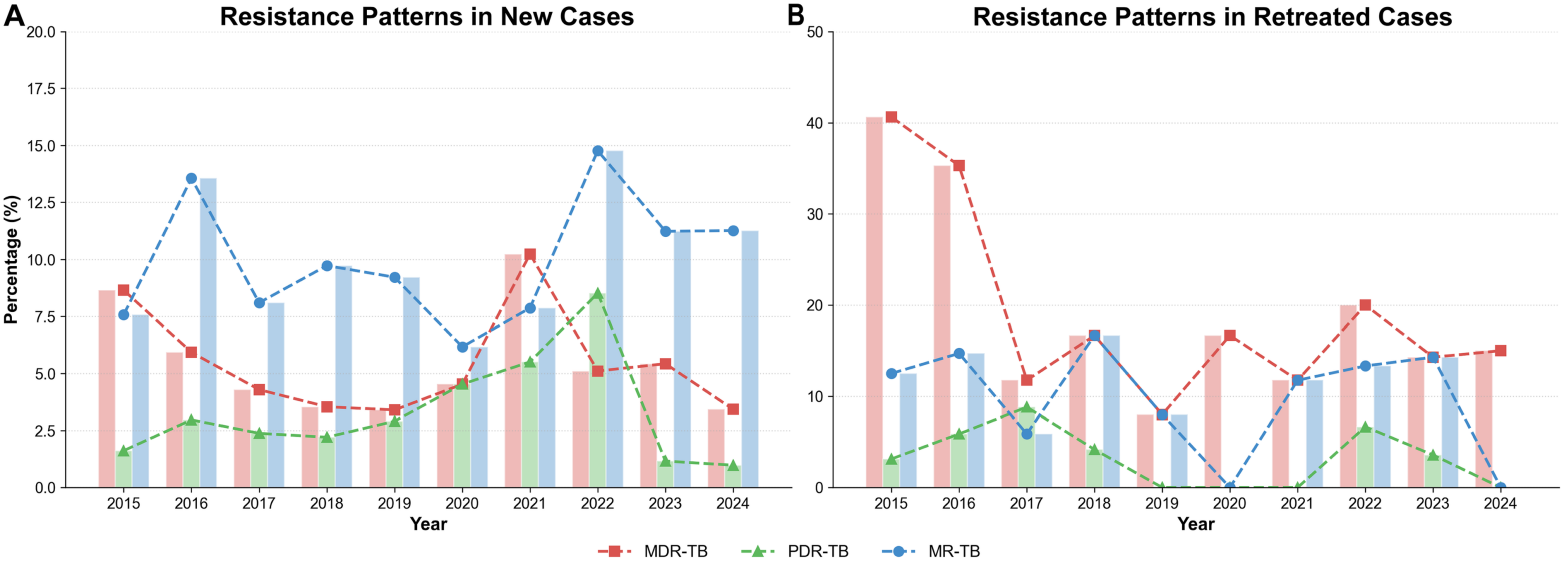
Annual trends in the proportions of drug resistance types among new and retreated TB patients, Lishui, China, 2015–2024. **(A)** Annual changes in MDR-TB, PDR-TB, and MR-TB proportions among new TB patients. **(B)** Corresponding patterns among retreated TB patients.

Among previously treated patients, the proportion of MR-TB decreased from 12.50% in 2015 to 0.00% in 2024, while that of PDR-TB fell from 3.13 to 0.00%; however, neither decline was statistically significant (MR-TB: *χ*^2^ = 0.565, *p* = 0.452; PDR-TB: *χ*^2^ = 0.861, *p* = 0.353). The proportion of MDR-TB declined from 40.63 to 15.00% (average annual reduction rate: 8.15%), marking it the only statistically significant downward trend observed in this group (*χ*^2^ = 6.961, *p* = 0.008). Notably, despite the downward trend, the MDR-TB proportion among previously treated patients remained as high as 15.00% in 2024, suggesting that the prevention and control situation in this population remains formidable (Table 5 and Figure **4**).

**Table 5:** Annual trends in drug resistance types among new and retreated TB patients.

Year	New case, *n* (%)					Retreated case, *n* (%)		
	No.	MDR-TB	PDR-TB	MR-TB	No.	MDR-TB	PDR-TB	MR-TB
2015	185	16 (8.65%)	3 (1.62%)	14 (7.57%)	32	13 (40.63%)	1 (3.13%)	4 (12.50%)
2016	236	14 (5.93%)	7 (2.97%)	32 (13.56%)	34	12 (35.29%)	2 (5.88%)	5 (14.71%)
2017	210	9 (4.29%)	5 (2.38%)	17 (8.10%)	34	4 (11.76%)	3 (8.82%)	2 (5.88%)
2018	226	8 (3.54%)	5 (2.21%)	22 (9.73%)	24	4 (16.67%)	1 (4.17%)	4 (16.67%)
2019	206	7 (3.40%)	6 (2.91%)	19 (9.22%)	25	2 (8.00%)	0 (0.00%)	2 (8.00%)
2020	243	11 (4.53%)	11 (4.53%)	15 (6.17%)	24	4 (16.67%)	0 (0.00%)	0 (0.00%)
2021	127	13 (10.24%)	7 (5.51%)	10 (7.87%)	17	2 (11.76%)	0 (0.00%)	2 (11.76%)
2022	176	9 (5.11%)	15 (8.52%)	26 (14.77%)	30	6 (20.00%)	2 (6.67%)	4 (13.33%)
2023	258	14 (5.43%)	3 (1.16%)	29 (11.24%)	28	4 (14.29%)	1 (3.57%)	4 (14.29%)
2024	204	7 (3.43%)	2 (0.98%)	23 (11.27%)	20	3 (15.00%)	0 (0.00%)	0 (0.00%)
	2071	108 (5.21%)	64 (3.09%)	207 (10.00%)	268	54 (20.15%)	10 (3.73%)	27 (10.07%)
*χ* ^2^		0.937	0.359	0.918		6.961	0.861	0.565
*P*-value		0.333	0.549	0.338		0.008	0.353	0.452

### 3.6 Sociodemographic determinants of MDR-TB risk

Occupation and prior treatment history emerged as statistically significant factors for MDR-TB in univariate screening (*p* < 0.05) and were subsequently entered into the multivariate logistic regression model. Although sex and age did not reach statistical significance in univariate screening (*p* > 0.05), they were retained in the multivariate model as *a priori* clinically relevant covariates to minimize residual confounding. Ethnicity and migrant status were excluded from the multivariate model due to both statistical non-significance in univariate analysis and low event counts in certain subgroups.

Multivariate analysis identified prior TB treatment history as the strongest independent risk factor for MDR-TB. Retreated patients had approximately 4.35 times the risk of MDR-TB compared with new patients (AOR = 4.345, 95% CI: 3.029–6.234, *p* < 0.001). Occupation was also identified as an independent risk factor, with farmers demonstrating a significantly higher risk of MDR-TB compared with non-farmer patients (AOR = 1.638, 95% CI: 1.047–2.562, *p* = 0.031) (Table 6).

**Table 6:** Risk factors for MDR-TB among new and retreated TB patients.

Characteristics	Non MDR-TB, *n* (%)	MDR-TB, *n* (%)	OR (95% CI)	*P*-value	AOR (95% CI)	*P*-value
Gender						
Female	542 (24.93)	32 (19.39)	1		1	
Male	1,632 (75.07)	133 (80.61)	1.380 (0.927–2.055)	0.134	0.861 (0.569–1.303)	0.479
Age (years)						
<45	630 (28.98)	49 (29.70)	1		1	
45–64	749 (34.45)	53 (32.12)	0.910 (0.612–1.354)	0.647	0.902 (0.616–1.321)	0.596
>64	795 (36.57)	63 (38.18)	1.020 (0.678–1.537)	0.933	1.209 (0.779–1.876)	0.397
Patient category						
New cases	1961 (90.17)	110(66.67)	1		1	
Retreated cases	213 (9.8)	55 (33.33)	4.603 (3.233–6.553)	<0.001	4.345 (3.029–6.234)	<0.001
Occupation						
Other	626 (28.79)	31 (18.79)	1		1	
Farmer	1,548 (71.21)	134 (81.21)	1.748 (1.170–2.612)	0.008	1.638 (1.047–2.562)	0.031
Nationality						
Han	2,140 (98.44)	162 (98.18)	0.858 (0.261–2.824)	1.000	–	
Others	34 (1.56)	3 (1.82)	1		–	
Migrant						
No	1712 (78.75)	135 (81.82)	1		–	
Yes	462 (21.25)	30 (18.18)	0.823 (0.547–1.239)	0.405	–	

## 4 Discussion

This study represents the first systematic retrospective analysis of first-line drug resistance patterns and trends in Lishui from 2015 to 2024, including 2,339 TB patients with pDST results. The overall prevalence of DR-TB was 20.09%, which is comparable to that of Wenzhou (20.41%), another prefecture-level city in Zhejiang Province (10). Regarding MDR-TB, the rate in Lishui (6.93%) was higher than that reported in Hangzhou between 2012 and 2022 (5.3%), and also exceeded the overall Zhejiang Province level (2.5% for new RR-TB cases and 4.3% for retreated RR-TB cases) (11, 12). The relatively high drug resistance rates in Lishui may be attributable to delayed healthcare-seeking associated with its mountainous geography and the high proportion of agricultural population, suggesting that regional differences in socioeconomic conditions and healthcare resource allocation are important factors influencing drug resistance levels. The MDR-TB rate among new patients in Lishui (5.21%) was slightly lower than the national average (6.9%) reported in a systematic review of new TB cases in China (15). whereas the MDR-TB rate among retreated patients (20.15%) was substantially higher than that of new patients, indicating that retreated patients represent a priority population for drug resistance prevention and control in Lishui and should be the focus of enhanced management interventions.

Comparison of baseline characteristics between the included cohort and the pDST-missing group revealed no significant differences in sex, age, occupation, or ethnicity (Supplementary Figure S1), suggesting that the excluded population did not differ substantially from the included population with respect to fundamental demographic variables. The higher proportions of retreated patients and migrants in the included cohort likely reflect the clinical practice of prioritizing DST for retreated cases and the greater accessibility of the municipal-level facility to urban-dwelling migrants. The identification of risk factors for MDR-TB, which relies on within-cohort case–control comparisons, is relatively robust to these compositional differences.

Regarding first-line drug resistance patterns, resistance rates in new patients ranked as INH > STR > RIF > EMB, consistent with the Wenzhou study (10), but differing from the Dalian study (INH > RIF > STR > EMB) (9). This discrepancy likely reflects regional differences in the genetic background of MTB strains and historical prescribing practices. Previous studies have confirmed substantial inter-regional variation in first-line drug resistance rates across Chinese provinces, further underscoring the necessity of localized drug resistance surveillance (16). In retreated patients, resistance rates ranked as INH > RIF > STR > EMB, consistent with findings from both the Wenzhou and Dalian studies (10). In terms of resistance categories, the proportions of DR-TB (33.96%) and MDR-TB (20.15%) in retreated patients were both significantly higher than in new patients (18.30 and 5.21%, both *p* < 0.001), which is consistent with previous studies (9, 12). This may be attributable to irregular drug use or inadequate treatment regimens during prior treatment courses, which create sustained drug pressure that facilitates the selection and accumulation of resistant mutants. Additionally, the proportion of MR-TB was similar between new and retreated patients (10.00% vs. 10.07%, *p* = 0.967). In retreated patients, repeated exposure to multiple treatment courses tends to drive resistance profiles toward multidrug resistance, which relatively reduces the proportion of MR-TB. The comparable MR-TB proportion in new patients suggests that mono-resistant strains may be circulating in the community, which warrants attention.

Over the 10-year period, the overall prevalence of DR-TB in Lishui declined from 23.50% in 2015 to 15.63% in 2024, though this trend did not reach statistical significance (APC: −1.30%) and was accompanied by substantial inter-annual fluctuations. Stratified analysis by treatment history indicated that this overall reduction was largely attributable to the marked decline among retreated patients, whose DR-TB prevalence fell from 56.25 to 15.00% (APC: −8.19%), consistent with the overall trends observed in previous studies from Wenzhou and Zhejiang Province (10, 12). The transient increase observed in 2021 and 2022 warrants specific consideration. The 2021 elevation should be interpreted with caution given the markedly reduced case count in that year, particularly within the retreated patient stratum. The more sustained spike in 2022, particularly among retreated patients, is more plausibly a delayed consequence of COVID-19-related service disruptions during 2020–2021. Interruptions to anti-TB drug supply, restricted patient mobility, and reduced clinic attendance during that period are well-documented contributors to treatment interruption (2), and incomplete or irregular treatment creates sustained drug pressure that selectively favors the emergence of resistant mutants (6). The subsequent return toward baseline levels is consistent with this interpretation, suggesting a transient, pandemic-related perturbation rather than a structural change in the local resistance landscape, a pattern also reflected in global DR-TB surveillance data for the same period (1). Meanwhile, DR-TB prevalence among new patients remained broadly stable over the study period (APC: +0.95%). The sustained improvement in drug resistance rates among retreated patients may be closely related to the continued implementation of DOTS and DOTS-Plus strategies, ongoing government funding for anti-TB treatment (including transportation subsidies and nutritional support), and progressively improved access to pDST. Furthermore, sustained government funding for local clinical research, comprehensive patient subsidies, and free DST has played a crucial role. In contrast, the relatively stable resistance rates in new patients suggest the persistence of drug-resistant strains in the community, which warrants further investigation through molecular epidemiological approaches. A systematic review of MDR-TB risk factors in low- and middle-income countries identified that reduced treatment adherence due to remote residence, low socioeconomic status, and increased travel distance to healthcare facilities during the COVID-19 pandemic are important contributors to the difficulty in suppressing primary drug resistance (17), and this international context is equally applicable to explaining the plateau in resistance rates among new patients in Lishui. Previous studies have demonstrated that primary transmission is a major driver of MDR-TB epidemics (18, 19). Therefore, in addition to continued strengthening of retreated patient management, efforts should be made to expand routine pDST for new patients, reduce diagnostic delays, and reinforce infection control measures.

In this study, prior TB treatment history was the strongest independent risk factor for MDR-TB, with retreated patients having approximately 4.35 times the risk of MDR-TB compared with new patients. This finding is highly consistent with a domestic meta-analysis (OR = 7.22) (20) and a Zhejiang Province study (OR = 10.9) (12), and the numerical differences may be attributable to variations in sample size, population composition, and study period across studies. This result is also consistent with international evidence, as prior treatment history has been identified as a key risk factor for MDR-TB across multiple studies in low- and middle-income countries (17, 21), likely because inadequate treatment exposes surviving strains to sustained drug pressure, facilitating the development of adaptive resistance mutations. These findings further confirm the role of standardized treatment in preventing acquired drug resistance, and retreated patients should be regarded as a priority population for local MDR-TB prevention and control.

Farmer occupation was also identified as an independent risk factor for MDR-TB in this study (AOR = 1.638, 95% CI: 1.047–2.562). Low socioeconomic status has received considerable attention in MDR-TB risk factor research. Farmers, constrained by limited financial resources and relatively poor access to healthcare, are more likely to experience treatment interruption or irregular drug use, thereby increasing the risk of drug resistance. A predictive model study of DR-TB patients in Wenzhou identified rural residence as an independent risk factor for MDR-TB (22), and Ichsan et al. (17) similarly reported that rural residence was an important predictor of MDR-TB treatment failure, further supporting the role of socioeconomic factors in the development of drug-resistant tuberculosis. These findings suggest that prevention and control strategies should give particular attention to the specific needs of rural and low-income populations, with enhanced adherence support and health education for these groups.

It is worth noting that sex, age, ethnicity, and migrant status did not reach statistical significance in this study, whereas age and sex have been identified as independent risk factors for MDR-TB in studies from multiple regions (10, 23), and population mobility has been linked to treatment interruption and elevated MDR-TB risk in prior studies conducted in China (19). These inconsistencies may reflect the relatively limited sample size in Lishui, local differences in population composition and disease epidemiology, and the potential mitigating effect of China’s nationwide TBIMS on treatment interruption risk among mobile patients, suggesting that risk factor profiles are region-specific and highlighting the importance of localized drug resistance surveillance and risk factor assessment.

This study has several limitations. First, the exclusion of culture-negative cases and those with missing pDST results may have introduced selection bias. Although comparison of baseline characteristics revealed broadly similar demographic distributions between included and excluded patients (Supplementary Figure S1), the excluded cases may still differ in unmeasured clinical characteristics, potentially affecting the generalizability of the findings. Second, due to testing capacity constraints, pDST was performed only for four first-line drugs (INH, RIF, STR, and EMB), with PZA and second-line drugs not included, precluding assessment of PZA resistance, second-line drug resistance, and XDR-TB trends, which limited the completeness of the drug resistance profile. Additionally, several clinical and socioeconomic variables were not collected, including smoking history, comorbidities, household income, and distance to healthcare facilities. Residual confounding by these unmeasured factors cannot be excluded, and future studies should incorporate such data for more comprehensive analyses.

## 5 Conclusion

This study systematically analyzed the drug resistance patterns and annual trends among TB patients in Lishui from 2015 to 2024. The overall prevalence of DR-TB showed a downward trend over the 10-year period, driven primarily by a marked decline in drug resistance among retreated patients, suggesting that prevention and control strategies targeting this group have achieved meaningful progress. The relatively stable resistance rates in new patients suggest ongoing circulation of drug-resistant strains in the community. Prior TB treatment history and farmer occupation were identified as independent risk factors for MDR-TB. Prevention and control authorities should prioritize retreated patients and socioeconomically vulnerable groups such as farmers, further promoting standardized treatment management and drug susceptibility testing to reduce the risk of MDR-TB in the local population.

## Data Availability

The raw data supporting the conclusions of this article will be made available by the authors, without undue reservation.

## References

[1] Organization, W.H. Global Tuberculosis Report (2025). Available online at: https://iris.who.int/handle/10665/383364 (Accessed June 10, 2026).

[2] Zhang G Yu Y Zhang W Shang J Chen S Pang X . Influence of COVID-19 for delaying the diagnosis and treatment of pulmonary tuberculosis-Tianjin, China. Front Public Health. (2022) 10:937844. doi: 10.3389/fpubh.2022.937844, 36530737 PMC9755169

[3] Tan EL Qin Y Yang J Li XJ Liu TQ Yang GB . Global burden of MDR-TB and XDR-TB: trends, inequities, and future implications for public health planning. BMC Infect Dis. (2025) 25:1225. doi: 10.1186/s12879-025-11566-2, 41039228 PMC12490063

[4] Guo Y Li J Lin L. Trends and forecast of drug-resistant tuberculosis: a global perspective from the GBD study 2021. Front Public Health. (2025) 13:1550199. doi: 10.3389/fpubh.2025.1550199, 40206160 PMC11979147

[5] Zhang L Ma X Gao H Bao C Wu Y Wu S . Analysis of care-seeking and diagnosis delay among pulmonary tuberculosis patients in Beijing, China. Front Public Health. (2024) 12:1369541. doi: 10.3389/fpubh.2024.1369541, 38689776 PMC11058192

[6] Liebenberg D Gordhan BG Kana BD. Drug resistant tuberculosis: implications for transmission, diagnosis, and disease management. Front Cell Infect Microbiol. (2022) 12:943545. doi: 10.3389/fcimb.2022.943545, 36211964 PMC9538507

[7] Xi Y Zhang W Qiao RJ Tang J. Risk factors for multidrug-resistant tuberculosis: a worldwide systematic review and meta-analysis. PLoS One. (2022) 17:e0270003. doi: 10.1371/journal.pone.0270003, 35709161 PMC9202901

[8] Xu P Li M Jiang Q Yang C Liu X Takiff H . Trends of drug-resistant tuberculosis in an urban and a rural area in China: a 10-year population-based molecular epidemiological study. Infect Drug Resist. (2024) 17:919–26. doi: 10.2147/IDR.S436563, 38481653 PMC10933515

[9] Pan Y Yu Y Lu J Yi Y Dou X Zhou L. Drug resistance patterns and trends in patients with suspected drug-resistant tuberculosis in Dalian, China: a retrospective study. Infect Drug Resist. (2022) 15:4137–47. doi: 10.2147/IDR.S373125, 35937782 PMC9348136

[10] Wu L Xia D Xu S Lin X Peng T Jiang X. Drug resistance patterns, trends, and risk factors for multidrug resistance of tuberculosis in Wenzhou, China: a ten-year retrospective analysis (2014-2023). Front Med. (2025) 12:1611322. doi: 10.3389/fmed.2025.1611322, 40740953 PMC12307396

[11] Li Q Zhao G Wu L Lu M Liu W Wu Y . Prevalence and patterns of drug resistance among pulmonary tuberculosis patients in Hangzhou, China. Antimicrob Resist Infect Control. (2018) 7:61. doi: 10.1186/s13756-018-0348-7, 29744042 PMC5930636

[12] Zhou L Wu B Huang F Liu Z Wang F Zhang M . Drug resistance patterns and dynamics of tuberculosis in Zhejiang Province, China: results from five periodic longitudinal surveys. Front Public Health. (2022) 10:1047659. doi: 10.3389/fpubh.2022.1047659, 36523585 PMC9745021

[13] Dheda K Mirzayev F Cirillo DM Udwadia Z Dooley KE Chang KC . Multidrug-resistant tuberculosis. Nat Rev Dis Primers. (2024) 10:22. doi: 10.1038/s41572-024-00504-2, 38523140 PMC13335523

[14] National Health Commission of the People’s Republic of China. China’s National TB Prevention and Control Technical Work Regulations (2020 Edition). Beijing: National Health Commission of the People’s Republic of China; (2020). Available at: http://tb.chinacdc.cn/ggl/202004/P020200414515703939844.pdf

[15] Jin C Wu Y Chen J Liu J Zhang H Qian Q . Prevalence and patterns of drug-resistant Mycobacterium tuberculosis in newly diagnosed patients in China: a systematic review and meta-analysis. J Glob Antimicrob Resist. (2024) 38:292–301. doi: 10.1016/j.jgar.2024.05.018, 38825149

[16] He GX Zhao YL Jiang GL Liu YH Xia H Wang SF . Prevalence of tuberculosis drug resistance in 10 provinces of China. BMC Infect Dis. (2008) 8:166. doi: 10.1186/1471-2334-8-166, 19077223 PMC2630916

[17] Ichsan I Redwood-Campbell L Mahmud NN Dimiati H Yani M Mudatsir M . Risk factors of MDR-TB and impacts of COVID-19 pandemic on escalating of MDR-TB incidence in lower-middle-income countries: a scoping review. Narra J. (2023) 3:e220. doi: 10.52225/narra.v3i2.220, 38450276 PMC10914066

[18] Yang C Luo T Shen X Wu J Gan M Xu P . Transmission of multidrug-resistant Mycobacterium tuberculosis in Shanghai, China: a retrospective observational study using whole-genome sequencing and epidemiological investigation. Lancet Infect Dis. (2017) 17:275–84. doi: 10.1016/S1473-3099(16)30418-2, 27919643 PMC5330813

[19] Liu D Huang F Li Y Mao L He W Wu S . Transmission characteristics in tuberculosis by WGS: nationwide cross-sectional surveillance in China. Emerg Microbes Infect. (2024) 13:2348505. doi: 10.1080/22221751.2024.2348505, 38686553 PMC11097701

[20] Feng M Xu Y Zhang X Qiu Q Lei S Li J . Risk factors of multidrug-resistant tuberculosis in China: a meta-analysis. Public Health Nurs. (2019) 36:257–69. doi: 10.1111/phn.12582, 30680796

[21] Zereabruk K Kahsay T Teklemichael H Aberhe W Hailay A Mebrahtom G . Determinants of multidrug-resistant tuberculosis among adults undergoing treatment for tuberculosis in Tigray region, Ethiopia: a case-control study. BMJ Open Respir Res. (2024) 11:e001999. doi: 10.1136/bmjresp-2023-001999, 38697676 PMC11086502

[22] Wu L Cai X Jiang X. Risk factors for multidrug-resistant tuberculosis: a predictive model study. Front Med. (2024) 11:1410690. doi: 10.3389/fmed.2024.1410690, 39399107 PMC11466792

[23] Wang Z Hou Y Guo T Jiang T Xu L Hu H . Epidemiological characteristics and risk factors of multidrug-resistant tuberculosis in Luoyang, China. Front Public Health. (2023) 11:1117101. doi: 10.3389/fpubh.2023.1117101, 37228738 PMC10203519

