# Supplementary material for: Drug-resistant tuberculosis in Lishui, China: first-line drug resistance patterns, trends, and risk factors from a 10-year retrospective study (2015–2024)
Source: Front Public Health. 2026 Jun 17;14:1855459. doi: 10.3389/fpubh.2026.1855459 (PMC13319091; doi:10.3389/fpubh.2026.1855459)
Supplement: Supplementary file 1 [file Data_Sheet_1.docx]

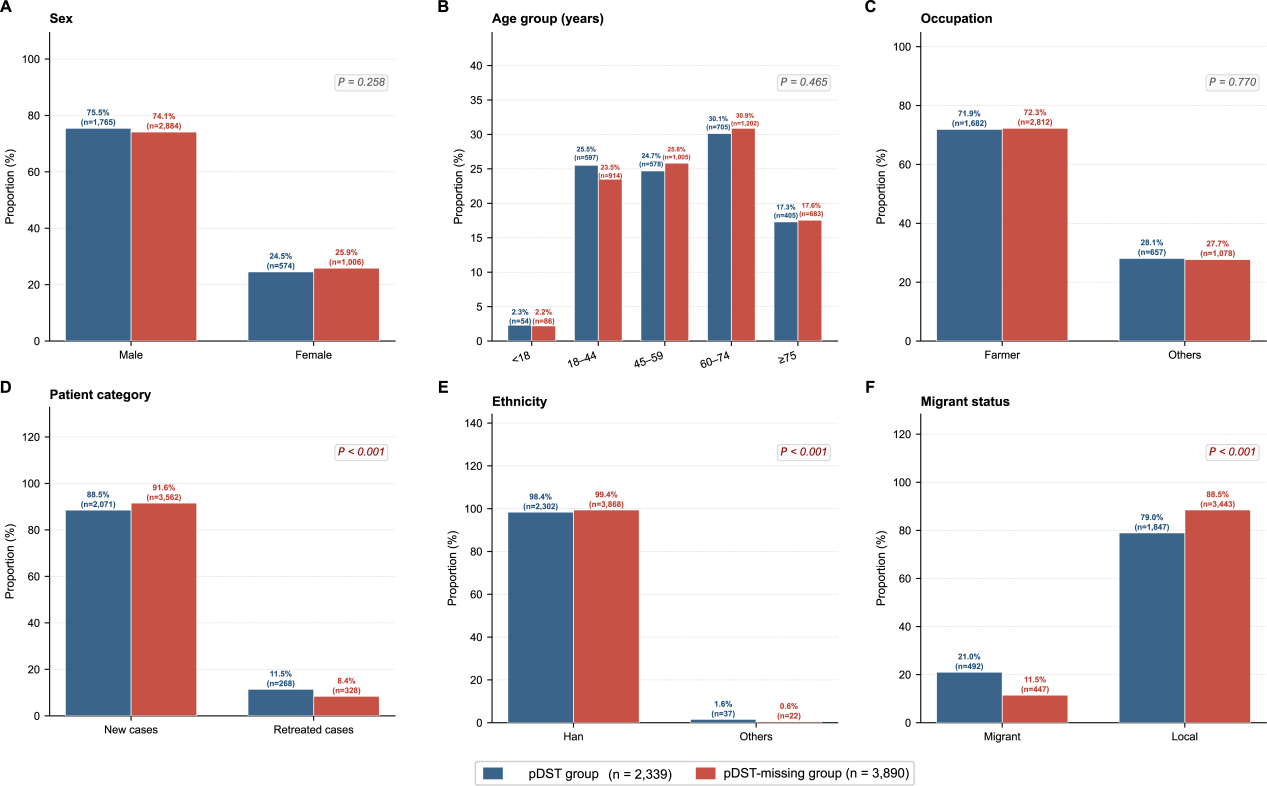


****Supplementary Figure S1.**** Comparison of baseline sociodemographic characteristics between the pDST group and the pDST-missing group, Lishui, China, 2015–2024. Bar charts illustrate the proportional distribution and absolute numbers of patients across six characteristics: (A) sex, (B) age group, (C) occupation, (D) patient category, (E) ethnicity, and (F) migrant status. Blue bars represent the included cohort (n = 2,339); red bars represent patients with missing or failed pDST results (n = 3,890). P values were derived from Pearson's χ² test.

**Supplementary Table S1.** Annual culture performance and pDST coverage among registered TB patients in Lishui, 2015–2024.

| Year | Cultures (n) | Negative (n) | Positive (n) | positivity rate (%) | pDST (n) | coverage rate (%) |
| --- | --- | --- | --- | --- | --- | --- |
| 2015 | 697 | 283 | 414 | 59.40% | 225 | 54.35% |
| 2016 | 785 | 319 | 466 | 59.36% | 277 | 59.44% |
| 2017 | 1018 | 389 | 629 | 61.79% | 284 | 45.15% |
| 2018 | 1190 | 499 | 691 | 58.07% | 289 | 41.82% |
| 2019 | 1019 | 380 | 639 | 62.71% | 255 | 39.91% |
| 2020 | 1042 | 349 | 693 | 66.51% | 287 | 41.41% |
| 2021 | 830 | 319 | 511 | 61.57% | 165 | 32.29% |
| 2022 | 972 | 300 | 672 | 69.14% | 253 | 37.65% |
| 2023 | 1144 | 370 | 774 | 67.66% | 321 | 41.47% |
| 2024 | 1027 | 287 | 740 | 72.05% | 276 | 37.30% |
| Total | 9724 | 3495 | 6229 | 64.06% | 2632 | 42.25% |
